# Tumour-associated macrophages as a potential target to improve natural killer cell-based immunotherapies

**DOI:** 10.1042/EBC20230002

**Published:** 2023-09-28

**Authors:** Takanori Kitamura

**Affiliations:** MRC Centre for Reproductive Health, The University of Edinburgh, Edinburgh, EH16 4TJ, United Kingdom

**Keywords:** cancer, immunotherapy, macrophage, natural killer cell, tumor microenvironments

## Abstract

Adoptive transfer of natural killer (NK) cells has been proposed as a novel immunotherapy for malignant tumours resistant to current therapeutic modalities. Several clinical studies have demonstrated that the NK cell-infusion is well tolerated without severe side effects and shows promising results in haematological malignancies. However, patients with malignant solid tumours do not show significant responses to this therapy. Such disappointing results largely arise from the inefficient delivery of infused NK cells and the impairment of their functions in the tumour microenvironment (TME). Tumour-associated macrophages (TAMs) are the most abundant stromal cells in the TME of most solid tumours, and a high TAM density correlates with poor prognosis of cancer patients. Although our knowledge of the interactions between TAMs and NK cells is limited, many studies have indicated that TAMs suppress NK cell cytotoxicity against cancer cells. Therefore, blockade of TAM functions can be an attractive strategy to improve NK cell-based immunotherapies. On the other hand, macrophages are reported to activate NK cells under certain circumstances.

This essay presents our current knowledge about mechanisms by which macrophages regulate NK cell functions and discusses possible therapeutic approaches to block macrophage-mediated NK cell suppression.

## Introduction

**Figure 1:**
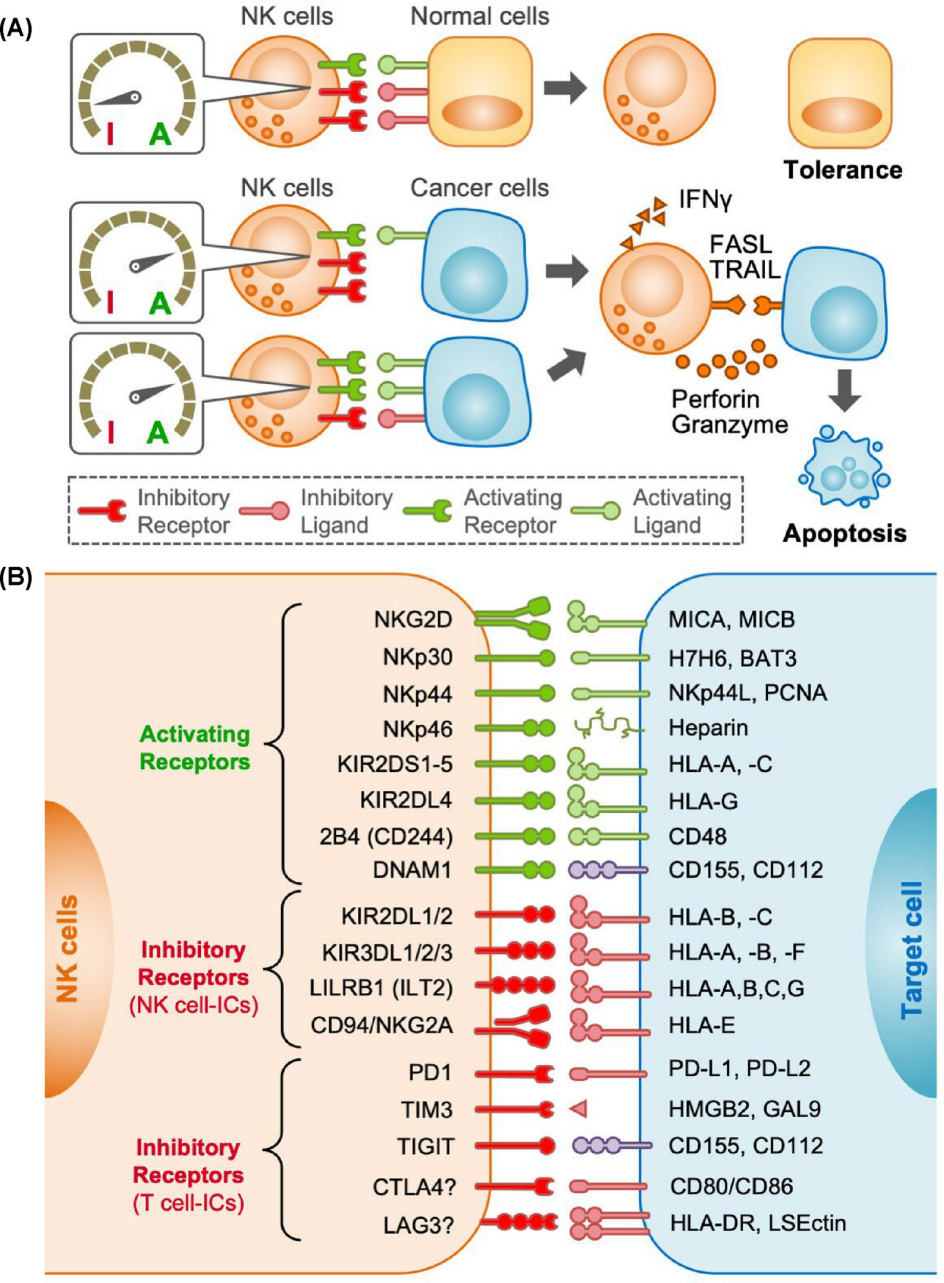
Regulation of NK cell responses against target cells (**A**) Natural killer (NK) cells express various receptors that transmit activation (A) or inhibitory (I) signals. NK cell response to target cells (being tolerant or triggering apoptosis) is determined by the overall levels of each signalling input. When target cancer cells reduce inhibitory ligands or increase activating ligands, activation signals overweigh inhibitory signals in NK cells. Activated NK cells induce apoptosis by secreting cytotoxic vesicles containing perforin and granzyme-B or presenting death ligands such as FASL and TRAIL. Activated NK cells also secrete interferon-γ (IFNγ) that induces apoptosis in certain cancer cells and regulates anti-immune reactions. (**B**) Canonical NK cell activating receptors (green) and inhibitory receptors (red) expressed by human NK cells and their ligands in target cells are shown. Potential inhibitory receptors known as T-cell immune checkpoints (ICs) are also shown. CD155 and CD112 (purple) can transmit both activation and inhibitory signals depending on the receptors. CTLA4 and LAG3 involvement in NK cell regulation is unclear and shown with ”?”.

Immunotherapies such as immune checkpoint inhibitors (ICIs) aim at boosting cytotoxic CD8^+^ T cell activation, which requires antigen presentation from targets via MHC class I (MHC-I) molecules. In many cases, however, malignant tumour cells reduce or lack MHC-I and/or tumour-specific antigens and avoid T cell attack. NK cells, however, do not require antigen presentation to kill targets. Instead, NK cell activation is regulated by a net balance of activating and inhibitory signals (Figure 1A). When inhibitory ligands are expressed in target cells whilst no or low activating ligands are expressed, inhibitory signals overwhelm activating signals, and thus NK cells do not attack them (tolerance). However, when inhibitory ligands are down-regulated (‘missing-self’) or activating ligands are up-regulated (induced-self) in target cells, NK cells are engaged to kill the targets via the secretion of cytotoxic vesicles containing perforin and granzymes, expression of death ligands such as Fas ligand (FASL) and tumour necrosis factor-related apoptosis inducing ligand (TRAIL), and production of interferon-γ (IFNγ) [1].

Therefore, the adoptive transfer of NK cells has been proposed as an alternative immunotherapy for cancer cells that evade T cell-mediated elimination [2]. Clinical trials have shown that NK cell infusion therapy is safe and effective for haematological cancers (e.g., leukaemia). However, no clear effects have been observed on solid tumours [3], which is believed to be primarily due to the presence of an immunosuppressive tumour micro-environment (TME) along with the inefficient delivery of therapeutic NK cells [4,5].

Solid tumours consist of a range of immune cells such as regulatory T cells, myeloid-derived suppressor cells, neutrophils, and macrophages that can suppress anti-tumour immune reactions [6,7]. Among these cells, macrophages are most abundant in most solid tumours, and high macrophage infiltration correlates with poor patient prognosis [8]. Moreover, numerous animal studies have identified that these tumour-associated macrophages (TAMs) are differentiated from monocytes recruited to the TME and promote tumour progression [9]. Since these recruited macrophages possess immunosuppressive phenotypes, they are considered a potential target for cancer immunotherapies [10]. Solid tumours, however, include macrophages derived from embryonic precursors (i.e., tissue-resident macrophages) that can contribute to antitumour immunity [11,12]. Furthermore, macrophages change their pro-inflammatory phenotype to anti-inflammatory or vice versa in response to environmental factors [13]. Such diversity and plasticity allow TAMs to regulate immune responses positively or negatively depending on the context. Consistent with this, contradictory effects of TAMs on NK cell functions are reported. For example, TAMs isolated from mouse mammary tumours impair the cytotoxicity of NK cells [14] and the higher TAM density correlates with a lower ratio of activated NK cells in human gastric cancer [15], which suggests NK cell suppressive roles for TAMs. In human liver cancer, however, NK cells in the peri-tumoral stroma where they co-localize with TAMs express an activation marker CD69 more frequently than NK cells in the TAM-sparse intra-tumoral region [16], which implies that TAMs promote NK cell function under certain circumstances.

This Essay discusses current understanding of macrophage effects on NK cell functions and potential approaches to prevent the macrophage-mediated NK cell suppression.

## NK cell activation by macrophages

The activation signal is transduced to human NK cells through natural killer cell protein group 2D (NKG2D) upon binding of its ligands MICA and MICB (RAE1 and MULT1 in mice), as well as via NKp30, NKp44, NKp46, activating killer cell immunoglobulin-like receptors (KIRs), 2B4, and DNAM1 that bind to different ligands [17,18] (Figure 1B).

It has been reported that tissue-resident (peritoneal) macrophages treated with poly I:C, a toll-like receptor (TLR) agonist, increase NK cell cytotoxicity against target cancer cells by increasing NKG2D expression on NK cells via secretion of IFNβ and IL15. Poly I:C also increases the expression of NKG2D ligands (RAE1, MULT1) in macrophages, which collectively enhance activating signals in NK cells [19]. Consistent with this result, poly I:C administration suppresses the metastatic growth of melanoma cells in mice by activating NK cells. In this model, alveolar macrophages from tumour-bearing mice treated with poly I:C promoted NK cell cytotoxicity *in vitro* [20]. Another study demonstrated that macrophages from the ascites of ovarian cancer patients significantly increased the NK cell-mediated cytolysis of ovarian cancer cells upon treatment with lipopolysaccharide (LPS) [21]. Similarly, human monocyte-derived macrophages (MDMs) activated by LPS promote proliferation, IFNγ secretion, and cytotoxicity of NK cells *in vitro*. Such macrophage-mediated NK cell activation requires CD48 expressed on macrophages and its receptor 2B4 expressed on NK cells [22]. It is also reported that co-culture of NK cells with human MDMs treated with LPS and IFNγ increases expression of activation markers (CD69 and CD107) and IFNγ in NK cells. Mechanistically, LPS and IFNγ increase CD48 expression on MDMs and promote IFNγ secretion from NK cells through 2B4. In addition, LPS/IFNγ stimulated MDMs produce IFNβ and IL23 and thereby enhance the expression of activating receptors NKG2D and NKp44 on NK cells. Moreover, LPS/IFNγ stimulated MDMs express high levels of IL15/IL15Rα membrane-bound complex, which also promotes NK cell activation [23].

**Figure 2:**
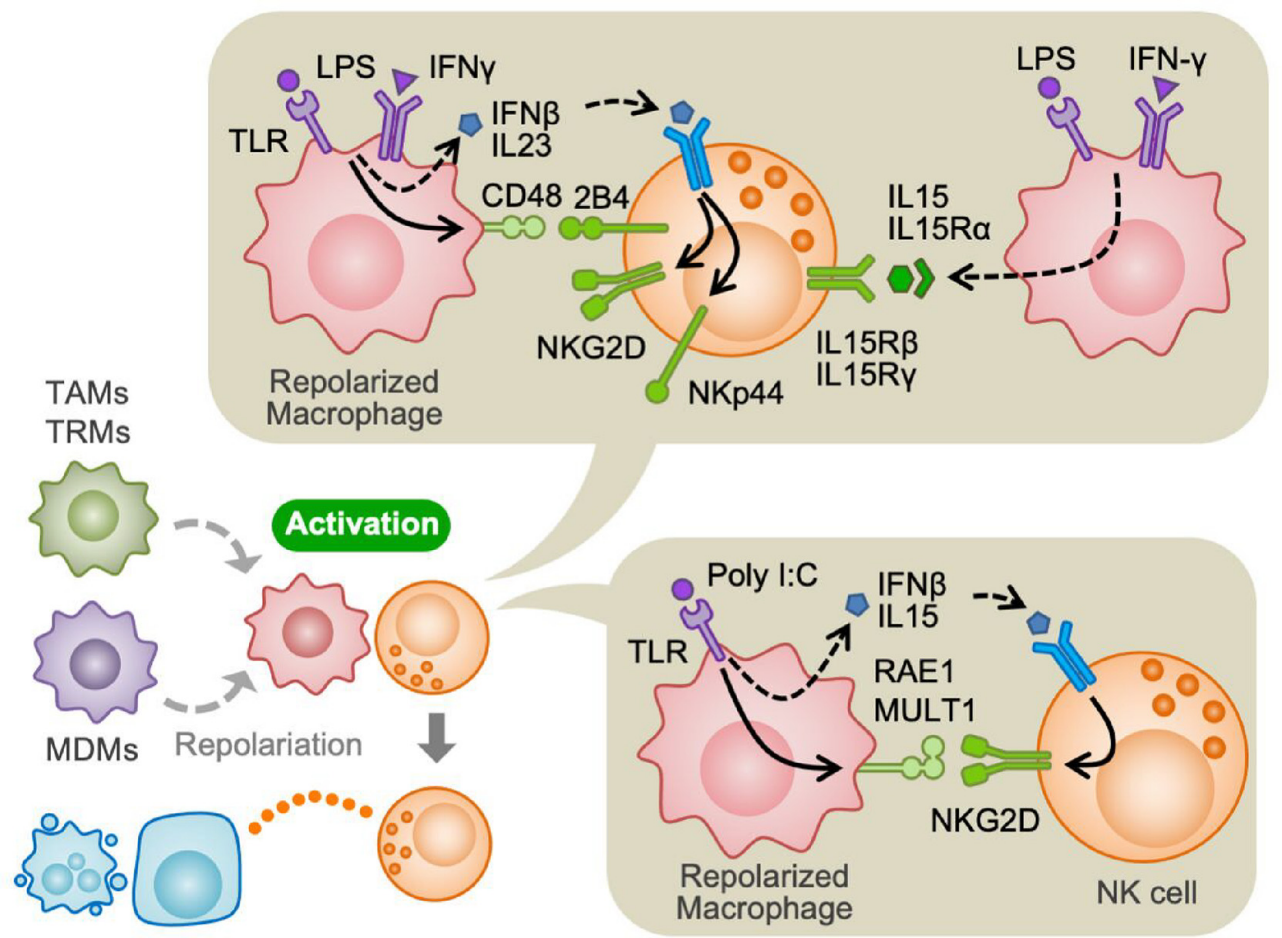
Activation of NK cells by macrophages Tumour-associated macrophages (TAMs), tissue-resident macrophages (TRMs) and monocyte-derived macrophages (MDMs) repolarize upon stimulation with IFNγ and/or toll-like receptor (TLR) ligands such as poly I:C and lipopolysaccharide (LPS). Repolarized macrophages activate NK cells by expressing activating ligands (RAE1, MULT1, CD48), inducing activating receptors in NK cells (NKG2D, 2B4, NKp44), and secreting IL-15. NK cells activated by macrophages increase cancer cell killing capability (indicated by an orange dot line). RAE1 and MULT1 are NKG2D ligands in mice.

Collectively, these data suggest that macrophages in TME can promote NK cell cytotoxicity when they are exposed to inflammatory stimuli such as TLR agonists, e.g., poly I:C, LPS, and/or IFNγ (Figure 2).

## NK cell inhibition by macrophages

### Persistent engagement of activating ligands

**Figure 3:**
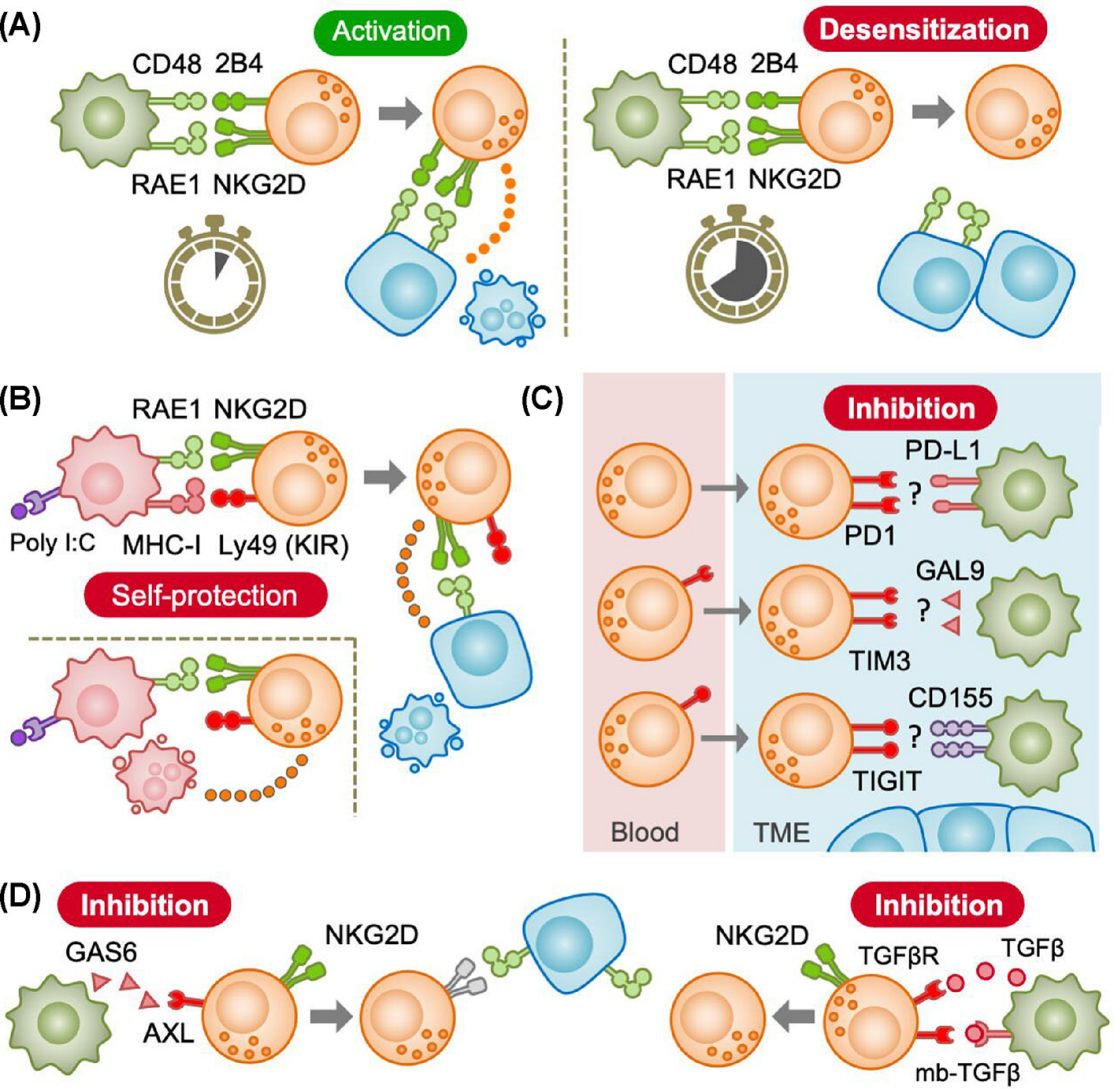
Suppression of NK cells by macrophages (**A**) Short-term interaction with TAMs expressing activating ligands (RAE1, CD48) promotes NK cell activation via activating receptors such as NKG2D and 2B4 (left). However, persistent engagement causes downregulation of activating receptors in NK cells and desensitizes NK cells (right). (**B**) Macrophages stimulated with poly I:C enhance NK cell cytotoxicity against cancer cells despite they express high levels of the inhibitory ligand, MHC class I (MHC-I). Thus, MHC-I expressed in macrophages may not inactivate NK cells persistently but protect themselves from NK cell killing. Ly49 is a mouse homologue of human inhibitory killer cell immunoglobulin-like receptor (KIR). (**C**) NK cells from cancer patients express canonical T cell immune checkpoint receptors such as PD1, TIM3, and TIGIT that suppress NK cell activation. Although TAMs can express their ligands (i.e., PD-L1, GAL9, CD155) depending on the tumour context, the extent to which they contribute to NK cell suppression remains unclear. (**D**) TAMs can produce growth arrest-specific gene 6 (GAS6) that inhibits NK cell activation through its receptors such as AXL. TAMs suppresses NK cell functions also by secreting transforming growth factor-β (TGFβ) or by expressing membrane-bound TGFβ.

It is reported that persistent engagement of activating ligands induces tolerance of NK cells [24,25], which could be a mechanism behind TAM-mediated NK cell suppression. In mice injected with melanoma cells, membrane NKG2D expression in tumour-infiltrating NK cells is downregulated whereas such a reduction is not found in *Rae1* knockout mice. In this model, TAMs increase RAE1 expression on their surfaces. Interestingly, sustained exposure (longer than 8 hours) to RAE1^+^ macrophages reduces NK cell activation induced by an agonistic NKp46 antibody, whereas such suppression is blocked by a recombinant NKG2D ligand. These results suggest that persistent engagement with NKG2D ligands on TAMs causes NKG2D down-regulation and subsequent desensitization in NK cells [26]. It is also reported that expression of NK cell activation markers (e.g., CD69, TRAIL, granzyme B) is increased by short-term (2 days) exposure to TAMs from human liver cancer, whereas long-term (8 days) exposure decreases. This macrophage-induced NK cell activation and subsequent dysfunction is attenuated by blocking 2B4 on NK cells [16]. Since prolonged contact with CD48^+^ cells internalizes membrane 2B4 expression in NK cells [27], it is likely that TAMs expressing activating ligands initially promote NK cell activation but lead to NK cell inactivation by persistent engagement (Figure 3A).

### Inhibition by immune checkpoint (IC) molecules

#### Canonical NK cell-ICs

The most characterized inhibitory receptors in human NK cells are inhibitory KIRs, leukocyte immunoglobulin-like receptors (LILRs), and NKG2A/CD94 that all bind to MHC-I [17,28]. They are known as canonical NK cell immune checkpoints (ICs) as they involve in NK cell tolerance [18] (Figure 1B).

A few studies have shown that a subset of TAMs express high levels of MHC-I (HLA-A) in human melanoma, liver cancer, and lung cancer [29–31]. However, MHC-I is not essential for suppressing NK cells in some *in vitro* models. For example, human NK cells co-cultured with MHC-I^high^ autologous monocytes do not impair their ability to lyse target cancer cells [32], and blockades of mouse MHC-I molecules or their receptors (Ly49) do not restore NK cell suppression induced by MHC-I^high^ bone marrow-derived macrophages (BMDMs) [33]. It is also reported that RAW264.7 mouse macrophage cell line expresses high MHC-I (H2-Qa1) upon stimulation with poly I:C, and these MHC-I^high^ macrophages enhance rather than suppress NK cell cytotoxicity. Interestingly, blockade of H2-Qa1 increases the NK cell-mediated macrophage lysis [19]. It is therefore possible that MHC-I on macrophages protect themselves from NK cell killing but may not inactivate NK cells persistently (Figure 3B).

#### Canonical T cell-ICs

Several ICs such as programmed cell death protein 1 (PD1), cytotoxic T-lymphocyte associated protein 4 (CTLA4), lymphocyte-activation gene 3 (LAG3), T-cell immunoglobulin and mucin domain 3 (TIM3), and T-cell immunoreceptor with immunoglobulin and ITIM domain (TIGIT) are known to regulate CD8^+^ T cell tolerance. However, their involvements in NK cell regulation are less understood.

PD1 expression is very low in naïve and activated NK cells from healthy donors but increased in NK cells from cancer patients [18]. In the lymph node of lymphoma patients, expressions of PD1 and its ligands PD-L1/PD-L2 are increased in NK cells and TAMs respectively. Interestingly, activation of PD-1^hi^ NK cells is suppressed by human PD-L1^hi^ MDMs, and this suppression is blocked by anti-PD1 antibodies [34]. CTLA4 competitively inhibits the binding of the co-stimulatory receptor CD28 and its ligands CD80/CD86, which is essential for T-cell activation. However, human NK cells do not express CTLA4 and are not co-stimulated by CD80 [35]. LAG3 is a receptor for MHC class II and LSECtin and regulates CD4^+^ and CD8^+^ T cell functions. However, blocking LAG3 does not affect human NK cell activity toward MHC-II^+^ target cells [36]. These data suggest minor or no involvement of CTLA4 or LAG3 in NK cell regulation. TIM3 is expressed by naïve NK cells from healthy donors and is upregulated in patients with melanoma, gastric cancer, and lung cancer [37–40]. It is reported that TIM3 cross-linking by antibodies suppresses human NK cell cytotoxicity [37], and TIM3 blockade reverses the impaired cytotoxicity or exhausted phenotype of patient-derived NK cells [39,40]. Interestingly, expression of HMGB1, a TIM3 ligand, is up-regulated in LPS-treated human MDMs and lactate-treated THP-1 macrophages [41,42]. Moreover, a subset of TAMs in invasive bladder cancer expresses another ligand galectin-9 (GAL9) [43]. However, some studies demonstrated that TIM3 ‘promotes’ rather than inhibits IFNγ secretion and cytotoxicity of human NK cells [44,45]. TIGIT is also expressed by NK cells in healthy donors and suppresses IFNγ production and cytotoxicity of NK cells upon engagement with its ligand CD155 [46–48]. In a mouse colon cancer model, TIGIT blockade prevents NK cell exhaustion and promotes NK cell-dependent tumour immunity [49]. Interestingly, TAMs from human colorectal cancer express high levels of CD155 compared with macrophages from adjacent normal tissues. Moreover, THP-1 macrophages reduce IFNγ and granzyme B expression of CD8^+^ T cells via a CD155 dependent mechanism [50], although their effects on NK cells are not investigated.

Taken together, current data suggest that TAMs may utilize signalling pathways mediated by PD1 and TIGIT to suppress NK cells in TME. However, further studies are necessary to define the role of these ICs in TAM-mediated NK cell regulation (Figure 3C).

### Inhibition by non-IC inhibitory molecules

A few molecules other than ICs are suggested to cause NK cell dysfunction. For example, sialic acid-binding immunoglobulin-like lectins (SIGLECs) and carcinoembryonic antigen-related cell adhesion molecule 1 (CEACAM1) are expressed by activated NK cells and their blockade enhances NK cell cytotoxicity against cancer cells [51,52]. However, it has not been investigated whether TAMs utilize these pathways to suppress NK cells. Receptors for growth arrest-specific gene 6 (GAS6), i.e., AXL, TYRO3, and MER, are also expressed by NK cells and are essential for their functional maturation [53]. Interestingly, GAS6 is reported to be expressed by TAMs in mouse models of breast and colon cancer [54] and inhibit IFNγ production and degranulation of NK cells *in vitro* by attenuating NKG2D activating receptor [55]. GAS6 is therefore an intriguing factor that may cause NK cell dysfunction within the TME. Another well-known NK cell inhibitory molecule is transforming growth factor β (TGFβ). For example, TAMs from human gastric cancer impair NK cell IFNγ expression, which is rescued by TGFβ blockade [15]. TAMs isolated from mouse mammary tumours and mouse BMDMs stimulated with IL-4 also inhibit NK cell activation by secreting TGFβ [14]. In a mouse model of metastatic breast cancer, TAMs from the tumour-bearing lung express membrane-bound TGFβ, and anti-TGFβ blocking antibody reverses TAM-mediated impairment of NK cell cytotoxicity [33]. As a potential mechanism, TGF reduces NKG2D surface expression on NK cells [56].

Collectively, these data suggest that TAMs constrain NK cell cytotoxicity by expressing GAS6 and/or TGFβ, although it remains unclear how much TAM-derived GAS6 contributes to NK cell suppression (Figure 3D).

## TAM targeting strategies that potentially improve NK cell efficacy

**Figure 4:**
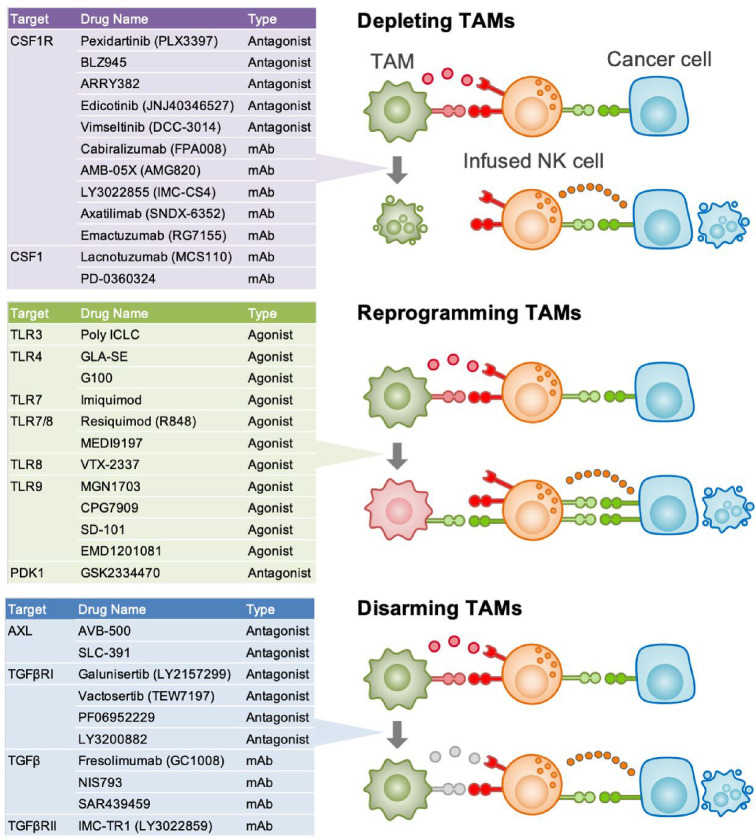
Current TAM-targeting strategies that potentially enhance NK cell function Anti-tumour efficacy of therapeutically infused NK cells could be enhanced by combining with TAM targeting, such as depletion, reprogramming, and disarming (i.e., blocking inhibitory ligands in TAMs). Tables show selective examples of compounds for each strategy that are currently tested in clinical trials as monotherapies or in combination with chemotherapeutics or immune checkpoint inhibitors. Note that effects of these compounds on the NK cell-based immunotherapies have not been investigated yet. CSF1R and TGFβR are the receptor for CSF11 and TGFβ, respectively.

Although several TAM-targeting strategies have been investigated in tumour context [10], their effects on the NK cell-infusion therapy remain unknown. Nevertheless, some pre-clinical studies suggest their potential to enhance endogenous NK cell functions (Figure 4).

### Depleting TAMs

Colony-stimulating factor 1 (CSF1) signalling is essential for the survival of most macrophage populations including TAMs, and thus CSF1 receptor (CSF1R) blockade reduces the number of TAMs in solid tumours. In a mouse breast cancer model, *Csf1r* knockout suppresses TAM accumulation and activates NK cells in metastatic tumours. Importantly, adoptive transfer of NK cells significantly reduces early metastatic tumour expansion only in TAM-depleted mice, suggesting that TAM depletion not only enhances intrinsic NK cell activity but also improves the efficacy of infused NK cells in suppressing metastatic tumour development [33].

Given pro-tumour functions of TAMs, several CSF1R antagonists have been tested as monotherapy or in combination with ICIs in some malignant tumours [10]. However, it is unknown whether these inhibitors improve NK cells’ anti-tumour functions in the TME. Importantly, a lung cancer mouse model demonstrated that a CSF1R inhibitor reduces the number of NK cells in the TME and enhances tumour metastasis [57]. This study suggests that CSF1R blockade may compromise NK cell functions as CSF1 signalling is essential not only for TAMs but also tissue-resident macrophages that can potentially support NK cells [58].

Another potential target is extracellular signal-regulated kinase 5 (ERK5) signalling since myeloid cell-selective knockout of ERK5 blocks TAM proliferation and metastatic tumour loads in a metastatic melanoma mouse model [59]. However, further studies are needed to determine whether NK cell functions are enhanced by TAM deletion via ERK5 blockade.

### Reprogramming TAMs

Macrophages alter their effects on NK cells depending on signals they receive. As described above (Figure 2), TLR signals prompt macrophages to activate NK cells. In a mouse model of squamous cell carcinoma, intra-tumoral injection of a TLR agonist induces repolarization of TAMs and suppresses tumour growth when combined with ICI treatment. NK cells contribute to the suppressive effect of this combination therapy whereas CD8^+^ T cells are also responsible for it [60]. Similar results were found in a mouse melanoma model, although NK cells are not involved in tumour suppression in this model [61].

Another potential target to reprogramme TAM is macrophage receptor with collagenous structure (MARCO), a scavenger receptor predominantly expressed by TAMs. Administration of anti-MARCO antibody induces repolarization of TAMs and suppresses tumour growth in mouse models of solid tumours [62]. In a mouse melanoma model, TAMs reprogrammed by anti-MARCO antibody activate NK cells to kill tumour cells via TRAIL [63].

3-Phosphoinositide-dependent kinase 1 (PDK1) is also a potential target since myeloid-selective knockout of PDK1 alters the phenotype of TAMs, enhances IFNγ expression in NK cells, and suppresses tumour growth in a mouse breast cancer model [64].

Although several TLR agonists and PDK1 inhibitors/antibodies are being tested in clinical trials, their therapeutic effects have not yet been reported [65,66]. Moreover, further pre-clinical studies are needed to determine whether these strategies can enhance NK cell infusion therapy efficacy.

### Disarming TAMs

Blocking NK cell suppressive molecules expressed by TAMs is another potential strategy. In mouse models of metastatic breast cancer and melanoma, treatment with a GAS6 receptor inhibitor markedly reduces tumour cell metastases through NK cell activation [67]. The administration of anti-Gas6 neutralising antibodies also promotes NK cell activation and suppresses metastatic tumour development in a mouse pancreatic cancer model [68]. However, their effects on TAM-mediated suppression of infused NK cells have not yet been clarified.

Since TGFβ signalling involves not only immune suppression but also malignant characteristics of tumour cells, several antibodies and inhibitors of TGFβ receptor have been developed and tested in clinical trials [69]. In a mouse breast cancer model, an anti-TGFβ antibody inhibits TAM-mediated NK cell suppression *in vitro*, and a selective TGFβ receptor antagonist partially but significantly suppresses early metastatic tumour growth when combined with NK cell infusion *in vivo* [33]. The combination therapy, however, failed to inhibit the late metastatic tumour expansion. It is thus possible that blockade of other NK suppressive molecules in addition to TGFβ might be required for TAM-targeting/NK cell-infusion combination therapy to eliminate established tumours.

## Future research directions

Current preclinical data suggest that combining TAM targeting with NK cell infusion could be a novel form of immunotherapy, although it has not been applied clinically yet. To facilitate the development of effective therapeutic approaches targeting TAM–NK cell interactions, it is crucial to address the following challenges.

- Most *in vitro* and pre-clinical studies on TAM–NK cell interactions utilize endogenous NK cells or freshly isolated NK cells that may not necessarily represent NK cells being infused in clinical trials. Recently, a technique for producing NK cells from human-induced pluripotent stem cells (iPSCs) has been established, which is considered a next-generation tool for NK cell infusion therapy. It will be imperative to investigate whether and how TAMs affect the efficacy of these therapeutic NK cells.
- Macrophages with different origins and activation status play distinct roles in immune regulation. Although RNA sequencing data have defined various TAM subsets with distinct transcriptomic profiles [70], their spatial localization in the TME or functions in the NK cell regulation are largely unknown. Emerging technologies such as spatial transcriptomics and multiplex immunostaining will facilitate answer these questions and thereby develop selective TAM targeting approaches.
- Macrophages stimulated with IFNγ and/or TLR ligands promote NK cell activation (Figure 2). These pro-inflammatory ligands, however, can also direct macrophages to express PD-L1 that inhibits anti-tumour immunity [71–73]. This highlights the need to investigate which factors in TME change macrophage functions in regulating NK cells by what mechanisms.
- Antibodies against PD1, PD-L1 or CTLA4 have achieved substantial success in the treatment of some cancers, but they are not effective in a large cohort of cancer patients. This prompted the investigation of other inhibitory pathways and led to the identification of novel immune checkpoint receptors such as TIM3 and TIGIT. Similarly, the simultaneous targeting of different immunosuppressive pathways will be required to improve the efficacy of NK cell-infusion therapy, and thus, the identification of novel inhibitory ligands expressed by NK cell-suppressive macrophages is critical.
- NK cell-derived cytokines impact dendritic and T-cell functions, promoting a conducive environment for tumour elimination [74,75]. While NK cells are also known to alter the macrophage phenotype in infectious diseases, little is known in tumour context [76,77]. Exploring the influence of endogenous and adoptively transferred NK cells on TAM function is crucial for developing therapeutics selectively targeting NK cell suppressive macrophage subsets.
- Cancer patients typically undergo chemotherapy or radiotherapy before trying immunotherapy. These conventional therapies have a negative impact on the survival of ‘intrinsic’ NK cells. However, they can increase tumour cell susceptibility to NK cells and create a favourable environment for “transferred” NK cells, which may benefit NK cell-infusion therapy [78,79]. Moreover, chemotherapy and radiotherapy can change the phenotype of TAMs to be immunostimulatory [80,81], although their effects on NK cell functions are unknown. Moreover, the response of the TME to chemotherapy and radiation could vary in individual cases. Therefore, it is important to define the effects of conventional treatments on TAM–NK cell interactions under different conditions (e.g., type of therapeutic, treatment schedule, tumour types) to design effective clinical trials. Advanced humanized mouse models supporting the engraftment of human macrophages and NK cells will greatly help answer this pertinent question.

## Summary

- TAMs constrain NK cells’ anti-tumour functions by downregulating activating receptors (NKG2D, 2B4) in NK cells or by expressing distinct immunosuppressive ligands such as PD-L1, GAL9, CD155, GAS6, and TGFβ.
- The TAMs’ NK cell-suppressive functions can be altered by targeting a distinct receptor or kinase such as TLR, MARCO, and PDK1.
- Blockade of TAM-derived immunosuppressive ligands such as GAS6 and TGFβ could inhibit NK cell dysfunction in the TME.
- TAM targeting is a prospective strategy for improving NK cell-based immunotherapies, although further research is required to find efficient targets for blocking TAM-mediated NK cell suppression.

## Open Access

Open access for this article was enabled by the participation of The University of Edinburgh in an all-inclusive *Read & Publish* agreement with Portland Press and the Biochemical Society under a transformative agreement with JISC.

## Abbreviations

BMDM: bone marrow-derived macrophage
CSF1: colony-stimulating factor 1
CSF1R: CSF1 receptor
CTLA4: cytotoxic T-lymphocyte associated protein 4
GAL9: galectin-9
GAS6: growth arrest-specific gene 6
IC: immune checkpoint
ICI: immune checkpoint inhibitor
IFNγ: interferon-γ
IL: interleukin
KIR: killer cell immunoglobulin-like receptor
LAG3: lymphocyte-activation gene 3
LILR: leukocyte immunoglobulin-like receptor
LPS: lipopolysaccharide
MARCO: macrophage receptor with collagenous structure
MDM: monocyte-derived macrophage
MHC-I: major histocompatibility complex class I
NK: natural killer
NKG2D: natural killer cell protein group 2D
PD1: programmed cell death protein 1
PDK1: 3-phosphoinositide-dependent kinase 1
TAM: tumour-associated macrophage
TGFβ: transforming growth factor-β
TIGIT: T-cell immunoreceptor with immunoglobulin and ITIM domain
TIM3: T-cell immunoglobulin and mucin domain 3
TLR: Toll-like receptor
TME: tumour microenvironment
TRAIL: tumour necrosis factor-related apoptosis inducing ligand
